# Population Genomic Signatures of Genetic Structure and Environmental Selection in the Catadromous Roughskin Sculpin *Trachidermus fasciatus*

**DOI:** 10.1093/gbe/evz118

**Published:** 2019-06-07

**Authors:** Yu-Long Li, Dong-Xiu Xue, Bai-Dong Zhang, Jin-Xian Liu

**Affiliations:** 1 CAS Key Laboratory of Marine Ecology and Environmental Sciences, Institute of Oceanology, Chinese Academy of Sciences, Qingdao, China; 2 Laboratory for Marine Ecology and Environmental Science, Qingdao National Laboratory for Marine Science and Technology, Qingdao, China; 3 Center for Ocean Mega-Science, Chinese Academy of Sciences, Qingdao, China

**Keywords:** population structure, local adaptation, *Trachidermus fasciatus*, habitat fragmentation, RAD-seq

## Abstract

Understanding the patterns of genetic diversity and adaptation across species’ range is crucial to assess its long-term persistence and determine appropriate conservation measures. The impacts of human activities on the genetic diversity and genetic adaptation to heterogeneous environments remain poorly understood in the marine realm. The roughskin sculpin (*Trachidermus fasciatus*) is a small catadromous fish, and has been listed as a second-class state protected aquatic animal since 1988 in China. To elucidate the underlying mechanism of population genetic structuring and genetic adaptations to local environments, RAD tags were sequenced for 202 individuals in nine populations across the range of *T. fasciatus* in China. The pairwise *F*_ST_ values over 9,271 filtered SNPs were significant except that between Dongying and Weifang. All the genetic clustering analysis revealed significant population structure with high support for eight distinct genetic clusters. Both the minor allele frequency spectra and *N*e estimations suggested extremely small *N*e in some populations (e.g., Qinhuangdao, Rongcheng, Wendeng, and Qingdao), which might result from recent population bottleneck. The strong genetic structure can be partly attributed to genetic drift and habitat fragmentation, likely due to the anthropogenic activities. Annotations of candidate adaptive loci suggested that genes involved in metabolism, development, and osmoregulation were critical for adaptation to spatially heterogenous environment of local populations. In the context of anthropogenic activities and environmental change, results of the present population genomic work provided important contributions to the understanding of genetic differentiation and adaptation to changing environments.

## Introduction

When different populations experience heterogeneous environments, local selection regimes can drive phenotypic divergence and modulate the underlying genomic architecture, thereby promoting local adaptation and ultimately initiating evolutionary diversification and speciation (Schluter 2000; Ferchaud and Hansen 2016). Detecting selection is an important step toward understanding the genetic basis of adaptive traits and the potential vulnerability or resilience of biodiversity to environment change (Brauer et al. 2016; Attard et al. 2018; Sandoval-Castillo et al. 2018). Reduction in genetic diversity can compromise the potential of a population to evolutionarily adapt to changing environments (Allendorf and Luikart 2007; Ouborg et al. 2010; Angeloni et al. 2012). Small and isolated populations are potentially faced with high extinction risks, due to the strong impact of genetic drift and inbreeding with low level of genetic variation (Frankham et al. 2002; Ouborg et al. 2010). Climate change and habitat loss are among the most important factors resulting in biodiversity decline (Barbosa et al. 2018). Evolutionary adaptations from standing genetic variation over generations are crucial for species to respond to the new or altered environments, leading to the expression of environment-dependent phenotypes (Barrett and Schluter 2008; Harrisson et al. 2014; Barbosa et al. 2018). Population with low level of genetic diversity is potentially faced with low effective population size (*N*e). *N*e is a key parameter to understand evolutionary processes and the viability of the overexploited and endangered populations as it determines the rate of genetic drift and inbreeding (Baalsrud et al. 2014). For population with low *N*e, the most important evolutionary forces are genetic drift and inbreeding, both leading to rapid loss of genetic diversity, which will result in a low-fitness risk of the population (McMahon et al. 2014). Estimates of *N*e can be theoretically useful to predict the impact of management practices on the loss of genetic variability due to the anthropogenic induced effects of random drift of the overexploited and endangered fishery species (Pita et al. 2017). For species long‐term persistence, there is a need to maximize genetic variability of species in order to maximize evolutionary potential. In addition, the long-term viability is also dependent on local adaptation, and genetic variability may be expected to decrease but would not limit long term evolutionary potential. Considering the importance of genetic diversity, it is essential and is the first step to appropriately define conservation units (CUs) to help guide management and conservation efforts (Allendorf and Luikart 2007; Funk et al. 2012). With the rapid development of high-throughput next-generation sequencing (NGS), genome-wide data make it easier to integrate information from neutral and adaptive loci to characterize CUs within a population genetics framework (Funk et al. 2012). Funk et al. (2012) proposed the delineation of three categories of CUs using genomic data: Evolutionarily significant units (ESUs) are defined using all the loci, maintenance of different ESUs will maximize evolutionary potential, management units (MUs) are defined using the neutral loci and are the basis for the short‐term management of populations, and adaptive units (AUs) are defined using the adaptive loci and are intended to help with protection of adaptive processes (Moritz 2004; Funk et al. 2012). Maintenance of different CUs will help maximize genetic variability of species in the face of environmental change.

The roughskin sculpin (*Trachidermus fasciatus* Heckel 1837), belonging to the family Cottidae, is a small carnivorous catadromous fish dwelling on the bottom of water (Wang 1999). *Trachidermusfasciatus* migrates from freshwater toward estuary and spawns in empty bivalve shells in winter, then the juveniles move upstream in spring to freshwater for growth (Shao et al. 1980; Onikura et al. 2002). When *T. fasciatus* migrates from freshwater toward estuary, it stops exogenous nutrition ingestion and its digestive organs show an involution in various degrees (Li et al. 1984). The thyroid gland of *T. fasciatus* changes seasonally in close relation to the seaward migration (Shao 1978). According to historical records, *T. fasciatus* was widely distributed along the coastlines of the Bohai Sea, the Yellow Sea, and the East China Sea as well as in rivers connected to seas (Wang and Cheng 2010). However, it has experienced severe population declines, habitat fragmentation and local extinctions in the past decades, probably due to human-mediated environmental change such as water pollution, dam construction, and blockage of migration channel (Wang and Cheng 2010). Besides, under the background of global climate change and human activities, runoff volume of some rivers (e.g., Dagu River-Qingdao, Luan River-Qinhuangdao, Wei River-Weifang) has been sharply reduced since recent decades, and drying-up of the river course has occurred frequently (Jiang and Wang 2013; Liu et al. 2013). This condition has seriously threatened the migration of *T. fasciatus*, and probably resulted in changes of life history of some *T. fasciatus* populations (e.g., Qingdao, Weifang, and Rongcheng population) from catadromous to noncatadromous. Considering its great biological, ecological, and economic importance in China, *T. fasciatus* had been included in the List of the Wildlife under Special State Protection as a second-class state protected aquatic animal by Chinese government in 1988.

In previous studies, researchers had studied population structure and estimated demographic parameters of *T. fasciatus*, mainly based on presumably neutral DNA-based markers (Xu et al. 2008; Zeng et al. 2012; Gao et al. 2013; Li et al. 2016). In our previous study, we found significant population structure and five genetic clusters among seven populations in China based on 16 microsatellite loci (Li et al. 2016). The five genetic clusters have presumably evolved from standing genetic variation of the same genetic lineage following deglaciation around 120,000 years ago (Gao et al. 2013). To date, few studies were conducted to estimate the genetic effective population size (*N*e) of *T. fasciatus*, likely due to the lack of power of genome-wide genotyping methods. Moreover, previous studies restricted to neutral markers can provide only limited insight to the mechanisms of local adaptation. Across its widely distributed range for *T. fasciatus*, the temperature, salinity, the levels of pollution, and pathogen conditions are largely different (Bao et al. 2002). Heterogeneous environment can result in patterns of localized adaptive divergence of populations (Savolainen et al. 2013; Sandoval-Castillo et al. 2018). In these circumstances, to improve the precision and accuracy of estimating a variety of important population genetic parameters and elucidating the mechanisms of local adaption of *T. fasciatus*, genomic tools as one promising approach may assist by increasing the number of variable genetic markers including both neutral and nonneutral loci. Restriction site-associated DNA tags sequencing (RAD-seq) is one of the rapid and cost-effective methods for genome-wide single nucleotide polymorphism (SNP) discovery and genotyping of nonmodel species without reference genomes (Catchen et al. 2013; Larson et al. 2014; McKinney et al. 2017).

In the present study, nine *T. fasciatus* populations were collected from its entire natural habitats in China. A comprehensive and high-resolution genome-wide assessment of population genetic status of *T. fasciatus* was conducted at both neutral loci and putative adaptive loci generated from RAD sequencing. We specifically aimed to address two questions that should have broad implications to conservation and management of *T. fasciatus*. First, do *T. fasciatus* exhibit fine genetic structure and what is the status of *N*e for different populations? Second, how the populations genetically adapted to local heterogeneous environments? The results of the present population genomic study can contribute to the understanding of genetic differentiation of aquatic species under anthropogenic activities and their adaptation to changing environments.

## Materials and Methods

### Sampling and RAD Sequencing

**Fig. 1.**
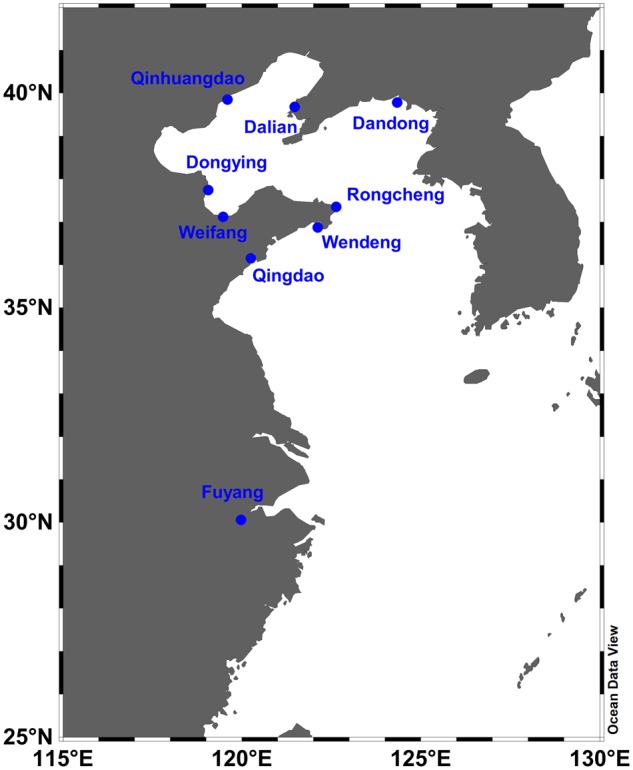
—Map of the sampling location for nine populations of *Trachidermus fasciatus*. Figure was generated by Ocean Data View 5.0.0 (Schlitzer, R., Ocean Data View, odv.awi.de, 2018).

A total of 202 individuals of *T. fasciatus* were collected from nine sites across its range-distribution in China during 2013–2017 (fig. 1 and supplementary table S1, Supplementary Material online). Tissues (including fin clips and muscles) were kept individually in 95% ethanol and stored at –80 °C. Genomic DNA samples were extracted following the standard phenol-chloroform extraction method. DNA extracts were visualized on 1% agarose gels to assess quality and were subsequently quantified using Qubit Fluorometer. Paired-end RAD (RPE) libraries were prepared followed the protocol as described by Etter et al. (2011). Briefly, genomic DNA was digested with the restriction enzyme *Eco*RI, and fragments were then ligated to Illumina P1 adapter with individual-specific index. Adapter-ligated fragments were subsequently pooled and randomly sheared, end repaired, dA added and ligated to Illumina P2 adapter. The fragments with both P1 and P2 adapter were enriched using high-fidelity PCR amplification. The RPE libraries were then sequenced on Illumina HiSeq machine at Novogene (Beijing, China).

### RAD Data Processing, Genotyping, and SNP Filtering

Raw reads from Illumina runs were demultiplexed into separated files according to individuals’ indexes. Reads with adaptors were removed using Cutadapt (Martin 2011), and further cleaned using “process_radtags” of Stacks 1.48 (Catchen et al. 2011) with a score limit of 13 and windows size of 0.1. Only those reads with sufficiently high sequencing quality, and that had an unambiguous RAD site, were retained. Finally, reads were processed using “clone_filter” of Stacks to remove potential PCR duplications.

RAD contigs were assembled using an optimized method as implemented in RADassembler (Li et al. 2018). This pipeline software uses Stacks to cluster reads from multiple individuals, and uses CAP3 (Huang and Madan 1999) to do local de novo assembly, which makes full use of the advantages of RPE reads. The optimal similarity thresholds for clustering within and across individuals were chosen using “chooseM” and “chooseN” functions of RADassembler. Reads were then aligned to the assembled contigs using BWA-MEM 0.7.15 (Li 2013) with default parameters, and were subsequently processed by SAMtools 1.7 (Li et al. 2009). SNP calling were performed using BCFtools in SAMtools. The generated SNPs were stored in a Variant Call Format (VCF) file.

In order to remove SNPs of low qualities, which might be resulted from false calling or paralogs, the following cut-offs were applied using in house Perl script (accessed at https://github.com/lyl8086/VCF_filter; Last accessed June 5, 2019): 1) only biallelic SNPs were retained; 2) SNP quality ≥ 30; 3) minimum coverage depth for an individual ≥ 6; 4) minimum genotype quality (GQ) for an individual ≥ 15; 5) minimum individual coverage for a population ≥ 12, that is at least 12 individuals were required to be genotyped for a SNP in each population; 6) total missing rate for all individuals of each SNP ≤ 0.1; 7) maximum observed heterozygosity (*H*_O_) for each population ≤ 0.5; 8) minor allele frequency (MAF) ≥ 0.05, but SNPs with MAF ≥ 0.2 in at least one population were retained. The final VCF file was converted into other formats necessary for subsequent analyses using PGDSpider 2.0.1.0 (Lischer and Excoffier 2012) and Plink 1.90b5.3 (Purcell et al. 2007). To minimize the effect of linkage disequilibrium (LD) for structure analysis, only one SNP with the lowest missing rate was retained for each RAD locus using in house Shell script and VCFtools 0.1.17 (Danecek et al. 2011).

### Genetic Diversity and Differentiation

Genetic diversity statistics within populations, including nucleotide diversity (*P*i), observed (*H*_O_) and expected heterozygosity (*H*_E_) and *F*_IS_, were estimated using “populations” in Stacks. Pairwise genetic differentiation (*F*_ST_) between populations and their significance were evaluated using Arlequin 3.5.2.2 (Excoffier and Lischer 2010) with 10,000 permutations. To further evaluate population subdivision, we performed an analysis of molecular variance (AMOVA) as implemented in Arlequin. To estimate differentiation among groups (*F*_CT_) and differentiation among populations within groups (*F*_SC_), three groups were defined considering both the geographic origin of populations (the Bohai sea, the Yellow sea and the East China sea) and geographic distance among them (see table 3 for grouping details).

**Table 3:** Analysis of Molecular Variance (AMOVA) Performed for Three Groups (“Dandong, Dalian, Qinhuangdao, Dongying and Weifang,” “Rongcheng and Wendeng, Qingdao,” “Fuyang”), and among All Populations

Source of Variation	Sum of Squares	Variance Components	Percentage Variation	Fixation Index
Among three groups	12,032.974	38.272	6.146	*F*_CT_ = **0.0615**
Among populations within three groups	11,613.018	32.707	5.252	*F*_SC_ = **0.0560**
Among nine populations	23,645.992	56.976	9.360	*F*_ST_ = **0.0936**

Note.—Significant *F*-statistic values were in bold (*P* <* *0.001).

Inference of population structure and individual assignment were carried out using three methods. Firstly, a model-based maximum likelihood method implemented in Admixture (Alexander et al. 2009) was used to estimate individual ancestries, which calculates estimates much more rapidly using a fast numerical optimization algorithm. The number of genetic clusters (*K*) was tested for values ranging from 1 to 10 with ten replications for each tested *K* value. In order to accurately estimate the true number of subpopulations, four supervised estimators (MedMedK, MedMeaK, MaxMedK, MaxMeaK) of Puechmaille (2016) were used to assess the most likely number of *K* implemented in StructureSelector (Li and Liu 2018). StructureSelector also used Clumpak (Kopelman et al. 2015) to generate plots for the selected *K*. Secondly, discriminant analysis of principal components (DAPC) method implemented in the R package Adegenet 2.1.0 (Jombart 2008) was used to visualize relationships among groups of samples and assign individuals to these groups. The alpha-score procedure was applied to select an appropriate number of principal components (PCs) as suggested by the manual. Thirdly, to visualize large and fine-scale patterns of population structure, NetView P 0.7.1 (Steinig et al. 2016) was used to visualize network relationships among all individuals with kNN step from 5 to 30. All the above structure analyses were performed on both all loci and neutral loci.

### *N*e Estimation and MAF Spectrum

Contemporary effective population size (*N*e) for each population was calculated with NeEstimator v2 (Do et al. 2014). This program implements three single-sample methods that based on LD, heterozygote excess, and molecular coancestry. Data sets with one SNP per locus were used to estimate *N*e, and three MAF limits (0.05, 0.01, and 0) were applied. Furthermore, in order to provide an illustration of the extent to which populations exhibit an L-shaped distribution, MAF spectrum for each population were plotted in R 3.5.0 (R Core Team 2018). MAF spectrum is expected to be L-shaped distribution due to the mutation–drift equilibrium (Nei et al. 1976). Deviations from an L-shaped distribution would indicate a population bottleneck (Luikart et al. 1998). In order not to bias allele frequency spectra, we did not set a lower minor allele threshold for the SNPs used in MAF calculation, the generated results were visualized in R.

### Outlier Detection, Annotation, and Delimitation of CUs

To identify putative loci under selection, two genome scan methods were applied. Firstly, the *F*_ST_-based method implemented in BayeScan 2.1 (Foll and Gaggiotti 2008) was used to identify high-*F*_ST_ outliers. BayeScan identifies outlier loci with a Bayesian model that decomposing *F*_ST_ coefficients into a population-specific component (beta) shared by all loci, and a locus-specific component (alpha) shared by all the populations using a logistic regression. BayeScan was performed using default parameters, and outlier loci were identified using a posterior odds of 10 with a 5% FDR. Secondly, a genotype–environment association method as implemented in Bayenv 2.0 (Coop et al. 2010) was used to identify putative loci correlated with environmental variations. Bayenv implements a Bayesian method that estimates the empirical pattern of covariance in allele frequencies between populations, and then uses this pattern as a null model for a test at individual SNPs. Four environmental variables were used for the tests of genotype–environment associations, including latitude, longitude, annual mean sea surface temperature (ASST) and mean sea surface temperature during catadromous period (CSST, from November to April, Wang et al. 2001). The high-resolution SST data (long term mean SST from 1971 to 2000) provided by the NOAA/OAR/ESRL PSD, Boulder, Colorado, USA, from their web site at https://www.esrl.noaa.gov/psd/ (last accessed June 5, 2019) was used. Because minor alleles may also be useful for identifying selection in naturally occurring organisms (Ahrens 2018), and alleles associated with environment can be significantly rarer than the common allele (Fournier-Level et al. 2011), we did not set a MAF cut-off on the data used in Bayenv tests (but a minimum of three copies of rare allele were required in at least one population in order to minimize genotyping errors). A Bayes Factor (BF) > 20 was required for the selection of outliers as evidence for “strong support,” following Kass and Raftery (1995). Three replicates were applied for Bayenv tests, and only those SNPs consistently screened out as outliers were retained.

Contig sequences containing outlier SNPs were then used for BLAST searches using local BLAST+ (Camacho et al. 2009) implemented in SequenceServer (Priyam et al. 2015) with an e-value of 1e–5. For the sequences with multiple matches, only the best hits were retained. Two reference sequences were used as the query database: Firstly, the complementary DNA (cDNA) sequences of *Gasterosteus aculeatus* from Ensembl (Zerbino et al. 2018), we chose it because the phylogenetic relationship between Cottales and Gasterosteales was close as described in Betancur-R et al. (2017) and the genome of *G. aculeatus* was well annotated; secondly, transcriptome reads of *T. fasciatus* from Ma et al. (2018) were de novo assembled into transcripts using Trinity 2.6.6 (Grabherr et al. 2011), and were then used as the database. Loci not matched to the first database were then searched against the second database (transcripts), and the aligned transcript sequences of *T. fasciatus* were then searched against the cDNA of *G. aculeatus*. The sequences with blasted hits were annotated using the UniProtKB database (The UniProt Consortium 2018), and were further categorized by a gene ontology (GO) term analysis implemented in Blast2Go (Conesa et al. 2005) with default parameters.

Three categories of CUs were defined based on different sets of loci. Firstly, the Delta K method (Evanno et al. 2005) was used to determine the most likely uppermost level of hierarchical population structure using the results of Admixture based on all loci for defining ESUs. A principal components analysis (PCA) was also performed to confirm the high-level genetic distinctiveness using GCTA (Yang et al. 2011). Secondly, MUs were defined according to the structure analysis results based on the neutral loci. Thirdly, to identify patterns of adaptive differentiation among populations, and to further define AUs, structure analysis was performed based on “adaptive loci.” We used only the high *F*_ST_ outliers as the putative adaptive loci following the method used in Barbosa et al. (2018), in which the high *F*_ST_ outliers were used to determine AUs. The high *F*_ST_ outliers were identified by BayeScan as being under directional selection. Inference of genetic clusters was carried using Admixture and the best *K* was accessed and visualized in StructureSelector.

## Results

### RAD Sequencing and Genotyping

RAD sequencing of 202 *T. fasciatus* individuals resulted in 1,448,111,215 read pairs, and 812,744,207 read pairs were retained after quality filtering. A total of 635,367,008 read pairs were discarded due to low quality, PCR duplications or ambiguous RAD tags. Using RADassembler, the optimal numbers of mismatch for clustering within and across individuals were set to 6 and 6, respectively, that is, *M* = 6 and *n* = 6 in Stacks. A total of 172,441 RAD loci were assembled and retained, with a mean contig length of 529 bp and N50 of 561 bp. After mapping read pairs of each individual to the generated RAD contigs, 2,057,173 SNPs were called. Of these, 13,681 SNPs passed the quality cut-off and were retained. The SNPs were with a mean depth of 57.65 and total genotyping rate of 93.93%. After retaining one SNP from each locus with the lowest missing rate, 9,271 SNPs were kept in a total of 202 individuals across nine sampling locations. These 9,271 SNPs (total genotyping rate = 94.13%) were used for downstream structure analysis.

### Population Genetic Diversity and Structure

Estimates of *H*_O_ and *H*_E_ average over the filtered 13,681 loci varied across the nine sampling locations (*H*_O_ = 0.1135–0.1543, *H*_E_ = 0.1039–0.1382, table 1). Nucleotide diversity (*P*i = 0.1066–0.1418) were similar to *H*_E_, and *F*_IS_ were not high across all the sites. Qingdao showed the lowest *H*_O_, *H*_E_, and *P*i, indicating the lowest genetic diversity among the nine populations. Rongcheng, Wendeng, Qingdao, and Fuyang displayed generally low level of percentage of polymorphic loci (46.69–58.48%), suggesting that lots of SNPs were monomorphic with one allele fixed, likely resulting from small populations after bottlenecks.

**Table 1:** Summary of Genetic Diversity Statistics for Nine Populations of *Trachidermus fasciatus*

Pop ID	Variant Sites	% Polymorphic Loci	Num Indv	*H*_O_	*H*_E_	*P*i	*F*_IS_	N*e*_LD_ (95% CI)
Dandong	13,681	70.2361	20.3856	0.1231	0.1175	0.1205	–0.0109	Infinite (∞)
Dalian	13,681	72.0050	23.4955	0.1304	0.127	0.1298	–0.0019	2591.1 (2062.7–3481.8)
Qinhuangdao	13,681	67.9629	20.1335	0.1543	0.1382	0.1418	–0.0439	32.0 (31.8–32.2)
Dongying	13,681	74.4609	21.5243	0.1350	0.1298	0.1329	–0.0084	512.8 (485.1–543.8)
Weifang	13,681	72.2973	20.8344	0.1374	0.1292	0.1324	–0.0186	313.9 (301.7–327.1)
Rongcheng	13,681	56.7356	20.8467	0.1192	0.1120	0.1148	–0.0152	23.8 (23.7–23.9)
Wendeng	13,681	58.4753	21.4057	0.1310	0.1167	0.1196	–0.0414	6.8 (6.8–6.9)
Qingdao	13,681	46.6852	20.5603	0.1135	0.1039	0.1066	–0.0236	100.3 (98.3–102.4)
Fuyang	13,681	57.0865	20.5565	0.1364	0.1313	0.1346	–0.0091	1766.0 (1391.6–2414.6)

Note.—Variant Sites, number of total SNPs; % Polymorphic Loci, proportion of SNPs in this populations; Num Indv, number of genotyped individuals averaged over all SNPs; *H*_O_, observed heterozygosity; *H*_E_, expected heterozygosity; *P*i, nucleotide diversity; *F*_IS_, inbreeding coefficient of individuals relative to the subpopulation; N*e*_LD_, effective populations size estimated based on LD method.

Most of the pairwise *F*_ST_ values over 9,271 SNPs between populations were significant (*P *<* *0.0014 after B-Y FDR correction), except that between Dongying and Weifang. *F*_ST_ ranged from –0.0038 between the two closest populations (Dongying and Weifang) to 0.1916 between Qingdao and Fuyang (table 2). The *F*_ST_ across all populations was 0.0936 and was statistically significant (*P *<* *0.001). The hierarchical AMOVA (table 3) showed strong and significant differentiation among the three groups of populations (*F*_CT_ = 0.0615, *P *<* *0.001).

**Table 2:** Pairwise *F*_ST_ Values Over 9,271 SNPs across Nine Populations of *Trachidermus fasciatus*

	Dandong	Dalian	Qinhuangdao	Dongying	Weifang	Rongcheng	Wendeng	Qingdao	Fuyang
Dandong	—	—	—	—	—	—	—	—	—
Dalian	**0.0405**	—	—	—	—	—	—	—	—
Qinhuangdao	**0.0592**	**0.0401**	—	—	—	—	—	—	—
Dongying	**0.0393**	**0.0335**	**0.0502**	—	—	—	—	—	—
Weifang	**0.0437**	**0.0259**	**0.0401**	–0.0038	—	—	—	—	—
Rongcheng	**0.1130**	**0.1108**	**0.1270**	**0.0980**	**0.1056**	—	—	—	—
Wendeng	**0.0988**	**0.1033**	**0.1199**	**0.0832**	**0.0895**	**0.0702**	—	—	—
Qingdao	**0.1358**	**0.1396**	**0.1574**	**0.1336**	**0.1424**	**0.1429**	**0.1077**	—	—
Fuyang	**0.1045**	**0.1283**	**0.1404**	**0.1213**	**0.1189**	**0.1764**	**0.1676**	**0.1916**	—

Note.—Values in bold were significant after FDR correction (*P *<* *0.0014).

**Fig. 2.**
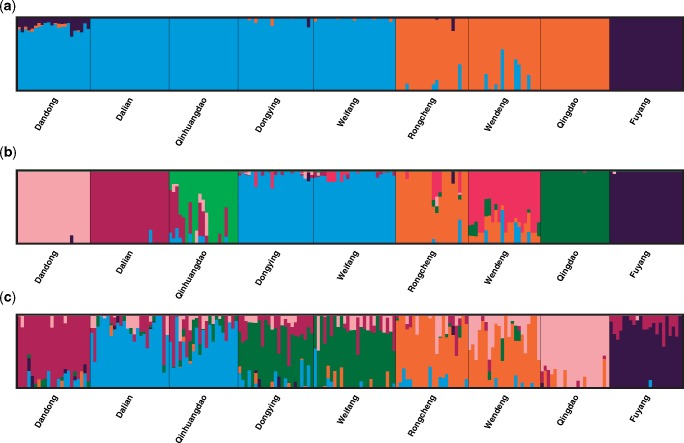
—Plots of the individual ancestry inference for (*a*) *K* = 3 based on all loci; (*b*) *K* = 8 based on all loci; and (*c*) *K* = 6 based on high-*F*_ST_ adaptive loci in nine populations of *Trachidermus fasciatus*.

**Fig. 3.**
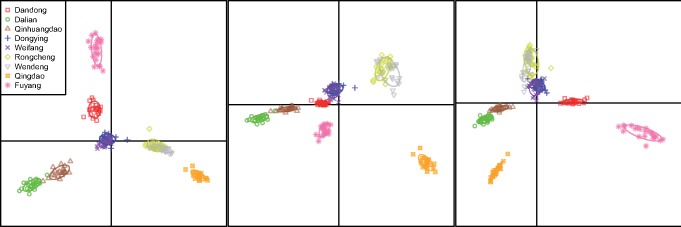
—DAPC scatter plots with prior population information. The plots were generated using 13 principle components with the first three coordinate axes based on all loci (explained 25.45% of the total variance).

The analysis using Admixture and StructureSelector indicated that there were highest supports for eight genetic groups (MedMedK, MedMeaK, MaxMedK, MaxMeaK = 8, supplementary fig. S1, Supplementary Material online) among individuals. The major mode for *K *=* *8 generated by Clumpak followed the patterns as indicated by the pairwise *F*_ST_ values: Dongying and Weifang formed into one group, and the other seven populations came out as distinct groups respectively (fig. 2*b*). DAPC analysis retained 13 PCs based on the alpha-score, which explained 25.45% of the total variance. The patterns of DAPC scatter plots were similar to the results revealed by Admixture, individuals of each population formed into distinct clusters respectively, except those from Dongying and Weifang (fig. 3), and several individuals of Rongcheng and Wendeng were overlapped. NetView P was able to reveal fine-scale clustering with kNN = 20 (supplementary fig. S2, Supplementary Material online), individuals were grouped into eight different clusters, with most individuals from Dongying and Weifang clustered together. Both Fuyang and Qingdao displayed little connections with other populations, suggesting that the two populations might be highly isolated, which also showed high *F*_ST_ values (> 0.1) in comparison with other populations.

### *N*e Estimation and MAF Spectrum

**Fig. 4.**
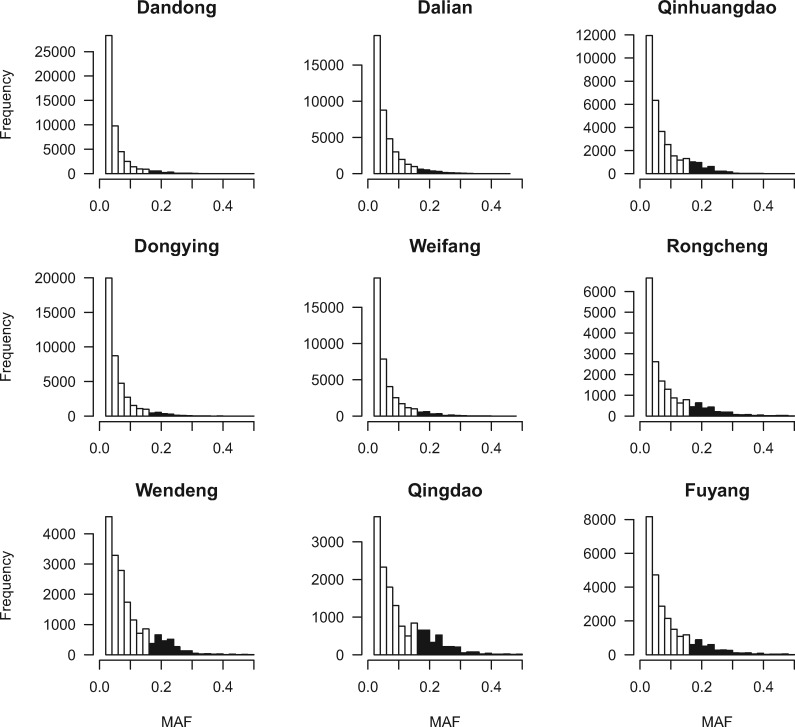
—SNP minor allele frequency (MAF) spectrum for nine populations of *Trachidermus fasciatus.* The *x*-axis represents categories of MAF with the corresponding allele counts on the *y*-axis. Color in black represents MAF > 15%.

Estimations of *N*e were varying across the sampling sites, and the MAF constraint had little effect on the values of *N*e, thus the results with no MAF cut-off were used. The *N*e ranged from 6.8 (Wendeng, 95% CI 6.8–6.9) to infinite (Dandong, 95% CI infinite; table 1). Rongcheng, Wendeng, and Qingdao displayed generally small *N*e, which might result from small population size and subsample effect (Kjeldsen et al. 2016). The estimations of *N*e were consistent with the pattern of genetic diversity, with Rongcheng, Wendeng, and Qingdao all showed low levels of *H*o, *H*_E_, and *P*i. MAF spectra generally exhibited L-shaped distributions, but varied markedly among populations (fig. 4). A certain number of rare alleles (> 15%) were observed in Qinhuangdao, Rongcheng, Wendeng, Qingdao, and Fuyang, suggesting small effective population size or newly founded populations. Specially, distributions of MAF spectra in Wendeng and Qingdao were much more distorted than the other populations, indicating potential bottlenecks with small *N*e. These results were consistent with the estimates of genome-wide *H*_E_, *P*i, and percentage of polymorphic loci within populations (table 1).

### Candidate Loci under Selection and Delimitation of CUs

BayeScan detected a total 56 outlier SNPs, of these 9 SNPs were potentially under balancing selection and 47 SNPs were potentially under divergent selection with posterior odds > 10 and FDR ≤ 0.05 (supplementary fig. S3, Supplementary Material online). To minimize false positives, only loci under divergent selection were used. Bayenv consistently identified 282 SNPs correlated with environmental variables after three independent replicates, of these 56 SNPs were associated with latitude, 18 SNPs were longitude related, 164 SNPs were ASST related, and 236 SNPs were CSST related. There were little overlaps between SNPs related with longitude and with other environmental variables (supplementary fig. S4, Supplementary Material online), whereas most of the ASST and CSST related SNPs were shared with each other (149 SNPs). Thereafter we used the combined results from BayeScan and Bayenv as the candidate adaptive loci, which corresponded to 313 SNPs from 300 RAD loci. Contig sequences of these loci were then used for further annotations.

Of the 300 candidate RAD loci, 69 were directly mapped to 59 cDNA of stickleback. A total of 136 of the remaining 231 loci were aligned to 135 transcripts of *T. fasciatus*, and 48 of these loci were further aligned to 48 cDNA of stickleback. In total, 117 of the 300 candidate RAD loci were aligned to 105 cDNAs (genes) of stickleback genome. These genes were found to be involved in diverse functions, and were putatively involved in local adaptive processes of *T. fasciatus*. Using Blast2Go, 74 loci were annotated and assigned to 138 GO terms. Most of the candidate loci containing SNPs putatively under selection could be categorized as having functions associated with catalytic activity, binding and hydrolase activity functions; cellular metabolic processes, biological regulation, development and response to simulation; or transport, signal transduction, cell communication and biosynthetic process (supplementary table S2, Supplementary Material online). These biological processes are potentially influenced by environmental variations like temperature and osmotic pressure.

**Fig. 5.**
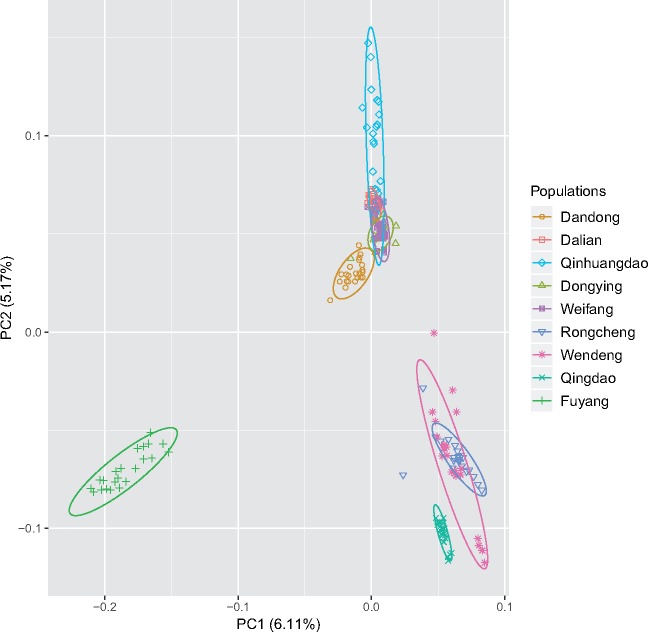
—PCA scatter plot using the first two principle components based on all loci.

Structure results based on neutral loci were similar to that on all loci (data not shown). Similarly, there was also high support for eight genetic clusters in nine populations of *T. fasciatus*, which displayed significant genetic divergence. The eight genetic clusters could be defined as eight MUs, including MU1 (Dandong), MU2 (Dalian), MU3 (Qinhuangdao), MU4 (Dongying & Weifang), MU5 (Rongcheng), MU6 (Wendeng), MU7 (Qingdao), and MU8 (Fuyang). The Delta K method could identify high-level genetic structure, which indicated the highest support for three major groups (*K* = 3, fig. 2*a*), the similar patterns were also indicated by PCA (fig. 5). Thereby, the three distinct genetic clusters could be defined as three ESUs: ESU1 (Dandong, Dalian, Qinhuangdao, Dongying, Weifang), ESU2 (Rongcheng, Wendeng, Qingdao), ESU3 (Fuyang), which should reflect the major divergence among populations of *T. fasciatus*. Using 47 high *F*_ST_ outlier SNPs, six genetic clusters were identified among individuals (*K* = 6, fig. 2*c*), corresponding to six AUs. Including AU1: Dandong, AU2: Dalian & Qinhuangdao, AU3: Dongying & Weifang, AU4: Rongcheng & Wendeng, AU5: Qingdao, AU6: Fuyang. The pattern of adaptive variation was similar to that using all loci (9,271 SNPs), except that Dalian & Qinhuangdao formed into one group and Rongcheng & Wendeng formed into another group. Levels of genetic divergence (*F*_ST_) between six clusters using outlier loci were listed in the supplementary table S3, Supplementary Material online. Among the six clusters, AU6 was the most distinctive one in terms of adaptive variation.

## Discussion

The present population genomic study delineated fine population genetic characteristics of the nine *T. fasciatus* populations by integrating data of putatively neutral and adaptive genetic variation at the genomic level. This is the first use of genome-wide SNP markers to assess a broad-scale population differentiation and signatures of selection in the *T. fasciatus*. Our study contributes to the growing body of literature identifying significant population structure and local adaptation in marine systems and has important implications for the conservation management of *T. fasciatus* and other endangered marine species.

### Causes of Population Structure and Low *N*e in *T. fasciatus*

The results provided a higher resolution of population structure compared that identified in our previous study based on microsatellite data (Li et al. 2016). In particular, in contrast to the microsatellite results, Dandong, Dalian, and Qinhuangdao populations were further genetically distinguished by RAD data sets. In addition, pairwise *F*_ST_ values were higher than those based on microsatellites. A good understanding of how ecological variants of fishes can impact their population structure will provide more comprehensive implications for conservation management and decision-making.

Habitat fragmentation is detrimental to species by reducing population size and genetic diversity and by restraining population connectivity (Lemer and Planes 2014; Young et al. 1996). Therefore, habitat fragmentation may have significant effects on population genetic structure because fragmentation usually leads to limited dispersal and thus lower connectivity levels among populations (Mossman and Waser 2001; Keyghobadi 2007; Lemer and Planes 2014). Because of its complex life history, the breeding and growth of *T. fasciatus* depends on different habitats including oceans, estuaries and rivers (Islam et al. 2007; Cao et al. 2009; Wang and Cheng 2010). In the past decades, the habitats of *T. fasciatus* has been markedly degraded and fragmented due to excessive anthropogenic activities (e.g., large-scale reclamation, pollution, dam construction, river flow modification), and the census populations size were sharply reduced (Wang and Cheng 2010). On the basis of our results, most populations were with low *N*e, thus are potentially faced with strong effect of genetic drift. Therefore, the divergence among the isolated populations may be caused by low gene flow and the effect of genetic drift among these populations due to habitat fragmentation. However, Dongying and Weifang populations are in close geographical proximity and may share the same spawning ground, which could result in high gene flow between the two populations.

In the presence of limited gene flow between fragmented populations, random genetic drift is expected to increase genetic differentiation among populations (Harrison and Hastings 1996; Lemer and Planes 2014). Currently, the populations of *T. fasciatus* have been severely declining due to the combination of overfishing and other anthropogenic activities. The strength of genetic drift is highly related with *N*e, which could be much lower than census abundance in marine species (Hauser et al. 2002; Pinsky and Palumbi 2014). Theoretical simulations suggest that short-term conservation of genetic diversity and inbreeding avoidance requires a *N*e > 500, and a safety balloon to maintain evolutionary flexibility in natural populations requires a *N*e > 1000 (Pita et al. 2017). The smaller *N*e estimates in Qinhuangdao, Rongcheng, Wendeng, and Qingdao indicated the exceptional status of these small populations. According to the MAF spectrum, the high proportion of rare alleles in Qinhuangdao, Rongcheng, Wendeng, and Qingdao also suggested of bottlenecks and strong genetic drift in these populations. Under the background of global climate change and human activities, runoff volume of some rivers as the migration channel of *T. fasciatus* (e.g., Dagu River for Qingdao population, Luan River for Qinhuangdao population, Wei River for Weifang population) has been sharply reduced since recent decades, and the drying-up of the river course has occurred frequently. Specially, the Rongcheng population is mainly distributed in Swan Lake. Swan Lake is a semienclosed coastal lagoon, the major environment perturbations might result in bottlenecks and small *N*e values, which will further strengthen genetic drift effects.

### Genomic Signatures of Environmental Selection in *T. fasciatus*

With the advantages of identification of genome-wide polymorphic markers, RAD sequencing can facilitate insight into studies of the ecological, evolutionary and conservation genomics (Andrews et al. 2016), especially in nonmodel organisms. However, RAD sequencing only covers a fraction of the total genome, and may have missed many loci under selection in local adaptation (Lowry et al. 2017). Nonetheless, despite the limited ability to detect adaptive loci, RAD sequencing remains a powerful tool for understanding the genetics of local adaptation in natural populations (Catchen et al. 2017). Genome scan methods, especially genotype–environment associations are still useful for uncovering candidate loci of relatively important ecological effect. In the present study, we did detect a number of important candidate loci that appear to be affected by spatially divergent selection, although some crucial adaptive loci might have been lost.

Environmental conditions can be important selective forces that shape the genotypic and phenotypic composition of local populations (Sanford and Kelly 2011). Environmental heterogeneity across different geographical locations may lead to phenotypic plasticity or genotypic responses (Hoffmann and Willi 2008). Aquatic organisms mainly exchange heat with their environment via conduction and convection, thus water temperature is one of the most important abiotic factor that influencing the phenotype and habitat of aquatic organisms (Angilletta 2009). The temperature of organism’s surrounding environment controls a variety of biological and physiological processes, including heat exchange (Kearney and Porter 2009), development (Kroeker et al. 2013), metabolic rates (Elliott 1976), responses (Batty et al. 1993), and aerobic scope (Pörtner and Knust 2007), which will further affect the higher functions (such as muscular activity, behavior, growth, and reproduction) and might thereby shape the long-term fate of species. We found evidence that a number of metabolic or developmental processes related genes (34 annotated candidate loci) were involved in the genetic adaptation of *T. fasciatus* to the local environment. One locus of interest is located in the *Glycerol-3-phosphate dehydrogenase (GPDH)* gene, which encodes enzyme that catalyzes the reversible redox conversion of dihydroxyacetone phosphate to sn-glycerol 3-phosphate. *GPDH* is a major link between carbohydrate metabolism and lipid metabolism, and is crucial in electron transport chain of the mitochondria. Indeed, *T. fasciatus* expends a vast amount of energy during the catadromous and reproduction processes. Previous physiological studies indicated that the digestive system of *T. fasciatus* was degenerative since its migration from freshwater to saltwater (Li et al. 1984), and the main energy sources were from intracorporal glycogen and lipid storage (Wang 1999). Thus, lipid and carbohydrate metabolism processes might play a crucial role in adaptation to environmental heterogeneity across different geographical populations, especially for the water temperature. Another candidate locus of interest was found in *the GLI family zinc finger 3 gene*, which could be related to embryonic organ development and dorsal/ventral pattern formation. This gene is also critical for specifying the fate of cortical neurons, and acts as the major negative transducer of the Sonic hedgehog (Shh) signaling pathway (Wang et al. 2011). The Shh pathway is one of the key regulators for animal development, suggesting that changes in body development of *T. fasciatus* could be implicated in the environmental temperature. This result is consistent with previous studies of *T. fasciatus*, for example studies on *T. fasciatus* suggested that high temperatures would restrict the body development (Wei et al. 1997), affect the growth rate of larvae (Shao et al. 1980). Therefore, genes related to metabolism and development were likely crucial candidates for the adaptation of *T. fasciatus* to water temperature. Studies in other marine organisms have also identified adaptive loci associated with temperature that were involved in similar diverse functions, examples including the greenlip abalone (Sandoval-Castillo et al. 2018), the Asian seabass (Wang et al. 2016), and the lake trout (Bernatchez et al. 2016).

Evidences of genetic adaptation in osmoregulatory related genes were also found. Among the annotated candidate loci, 19 loci were categorized as having functions involved in iron binding, including the metal iron binding, cation binding, etc., which might be involved in osmoregulation. As a catadromous fish, *T. fasciatus* have to uptake ions from the environment to compensate for salts lost in fresh water, whereas in saltwater they have to diffuse ions to compensate for the passive diffusion of salts into their bodies. Importantly, during spawning migration, *T. fasciatus* actively migrate from freshwater to saltwater to uptake sufficient iodine for the development of thyroid and sexual maturity (Shao 1978; Shao et al. 1980). The osmoregulatory and ion-exchange related genes may play a key role in adaptation to freshwater and saltwater systems. Indeed, some populations of *T. fasciatus* might not be able to complete the migration between seawater and freshwater due the lack of connections with rivers or drying-up of the rivers. Thus these populations (e.g., Weifang, Rongcheng, and Qingdao) might have been lived completely in saltwater, which has been confirmed by otolith microchemistry analysis (Bi 2013). It is possible that there may be a difference of iron regulation between these populations and other populations with complete catadromous life history. Interestingly, we did find the candidate adaptive loci in the solute carrier family 39 (SLC39) like and Cation/H+ exchanger like gene, which were involved in osmoregulation. SLC family genes are multifunctional and are often involved in acid–base balancing via movement of monovalent and divalent anions (Willoughby et al. 2018). Previous studies in stickleback and trout also identified similar gene (SLC26) being involved in adaptation to the freshwater–saltwater systems (Hohenlohe et al. 2010; Willoughby et al. 2018). The documentation of genes involved in both metabolic and osmoregulatory pathways provides the first glimpse of how *T. fasciatus* have been adapted to the spatially varying environment of local populations.

Several candidate loci were annotated as having functions involved in biological regulation, response to stimulus, immune and signaling processes. For instance, one locus was found in the *Transporter 1, ATP-binding cassette, subfamily B gene (TAP1)*, which was involved in MHC protein binding, antigen processing and presentation of exogenous peptide antigen. TAP1 functions as the membrane transporter associated with antigen processing that are involved in transport of peptides that bind to MHC molecules (Flajnik and Kasahara 2001), and plays a major role in transportation of foreign peptides through the endoplasmic reticulum for processing before loading onto MHC molecules (Elgert 2009). Actually, immune response was also shown to be related to the local adaptation to different water temperature in pipefish (Flanagan et al. 2016) and abalones (Sandoval-Castillo et al. 2018). Overall, these findings suggested that the observed adaptive divergence of *T. fasciatus* might involve diverse functional changes related to spatially varying environments. This information about the functions of annotated candidate loci could help us to understand the genetic basis of adaptation to important environmental variables like temperature, and provided useful leads for future research in marine species.

### Conservation and Management Implications for *T. fasciatus*

Our study used population genomic techniques to offer high resolution genetic information to improve conservation and management policies and decisions, which previously were not clear with respect to patterns of adaptive genetic variation. The results presented here would provide complementary information for management, especially in defining CUs and setting conservation priorities and management plans for *T. fasciatus*.

The results demonstrated that the nine populations of *T. fasciatus* should be defined as eight MUs (based on neutral loci), six AUs (based on adaptive loci), and three ESUs (based on all loci). CUs with lower genetic diversity and *N*e require high conservation priority, such as MU7 (Qingdao) & MU8 (Fuyang) or AU5 (Qingdao) & AU6 (Fuyang), which show high level of isolation with other populations. In particular, MU6 (Wendeng) deserves special attention given the lowest *N*e, which is related to low fitness risk. However, the census population size of MU6 might be relative large, which could be much larger than that of MU8 (Wang and Cheng 2010), but the *N*e of MU6 were much smaller than that of MU8. A large population census size with an extremely low genetic variation and *N*e could be attributed to overharvest or natural bottlenecks (Pita et al. 2017). Overharvest and habitat fragmentation due to anthropogenic activities can lead to the loss of genetic variation and a consequent reduction in evolutionary potential and adaptive ability (Hauser et al. 2002; Pinsky and Palumbi 2014). Although there is no known targeted fishery for this species, *T. fasciatus* is often caught as bycatch from bottom-trawl fishery and trap net fishery in China (Wang and Cheng 2010). As a ferocious carnivorous fish with large food consumption, *T. fasciatus* needs to live in area with plenty of food resources. The various type of habitats (inshore sea area, estuaries, and rivers) for the breeding and growth of *T. fasciatus* also suggested its sensitive to environmental changes. Currently it is difficult for *T. fasciatus* to survive, migrate, breed, and completed the entire life cycle (Wang and Cheng 2010; Li et al. 2016). Thus, carrying out prohibited fishing areas and prohibited fishing periods as well as habitat restoration and protection are of great concerns and are probably the most immediate action to take at this time to protect *T. fasciatus*.

Another government-promoted conservation action on *T. fasciatus* was stock enhancement (Bell et al. 2006). A large number of evidences indicated that stock enhancement could improve the abundance of target species, but it might also impose some risks on the local populations (e.g., mixing genetic lineages, spreading pathogens). For the management and conservation of this species, the eight distinct clusters detected in the present study should be considered as different MUs. Furthermore, *T. fasciatus* that were recruited within their local adaptive cluster (AUs) might show higher fitness (with regard to variation in the thermal environment and migration routes) than recruited in clusters found elsewhere.

## Conclusions

By analysis of genome-wide data of nine *T. fasciatus* populations, fine population genetic structure and genomic signatures of adaptations across the species’ range were detected. Genetic clustering analysis revealed significant population structure with high support for eight distinct clusters among nine populations. The strong genetic structure could be attributed to habitat fragmentation and genetic drift due to anthropogenic activities. A substantial number of genetic variants appeared to be under differential selective pressure across the range of *T. fasciatus*. Annotations of candidate adaptive loci suggested that the genes involved in diverse functions like metabolism, development and osmoregulation are critical for the adaptation to environmental heterogeneity across the species’ range.

## Supplementary Material

Supplementary data are available at *Genome Biology and Evolution* online.

## Supplementary Material

Supplementary_Material_evz118

## Notes

**Data deposition:** This project has been deposited at the NCBI Nucleotide Short Read Archive (SRA) under the accession No. PRJNA360511.
