# Supplementary material for: Population Genomic Signatures of Genetic Structure and Environmental Selection in the Catadromous Roughskin Sculpin Trachidermus fasciatus
Source: Genome Biol Evol. 2019 Jun 7;11(7):1751–64. doi: 10.1093/gbe/evz118 (PMC6601870; doi:10.1093/gbe/evz118)
Supplement: Supplementary_Material_evz118 [file supplementary_material_evz118.docx]

# Figures

Fig. S1 Plot of MedMedK, MedMeaK, MaxMedK, MaxMeaK values generated by StructureSelector for Admixture runs based on all loci. All the four parameters supported eight genetic clusters.


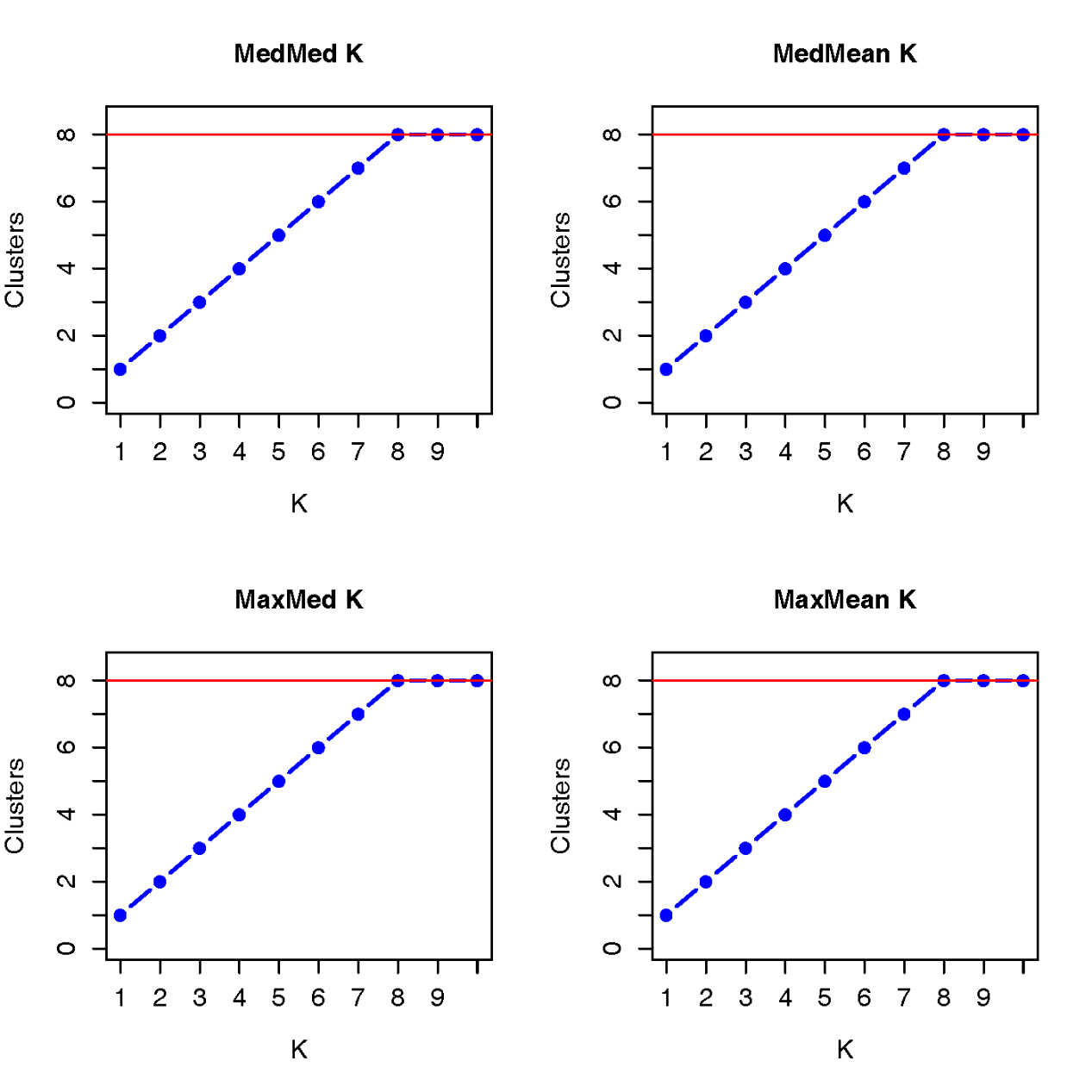


Fig. S2 Individuals clustering for nine populations of *T. fasciatus* constructed by NetView P, kNN = 20 was used. Figure was generated by Cytoscape (Shannon, et al. 2003).


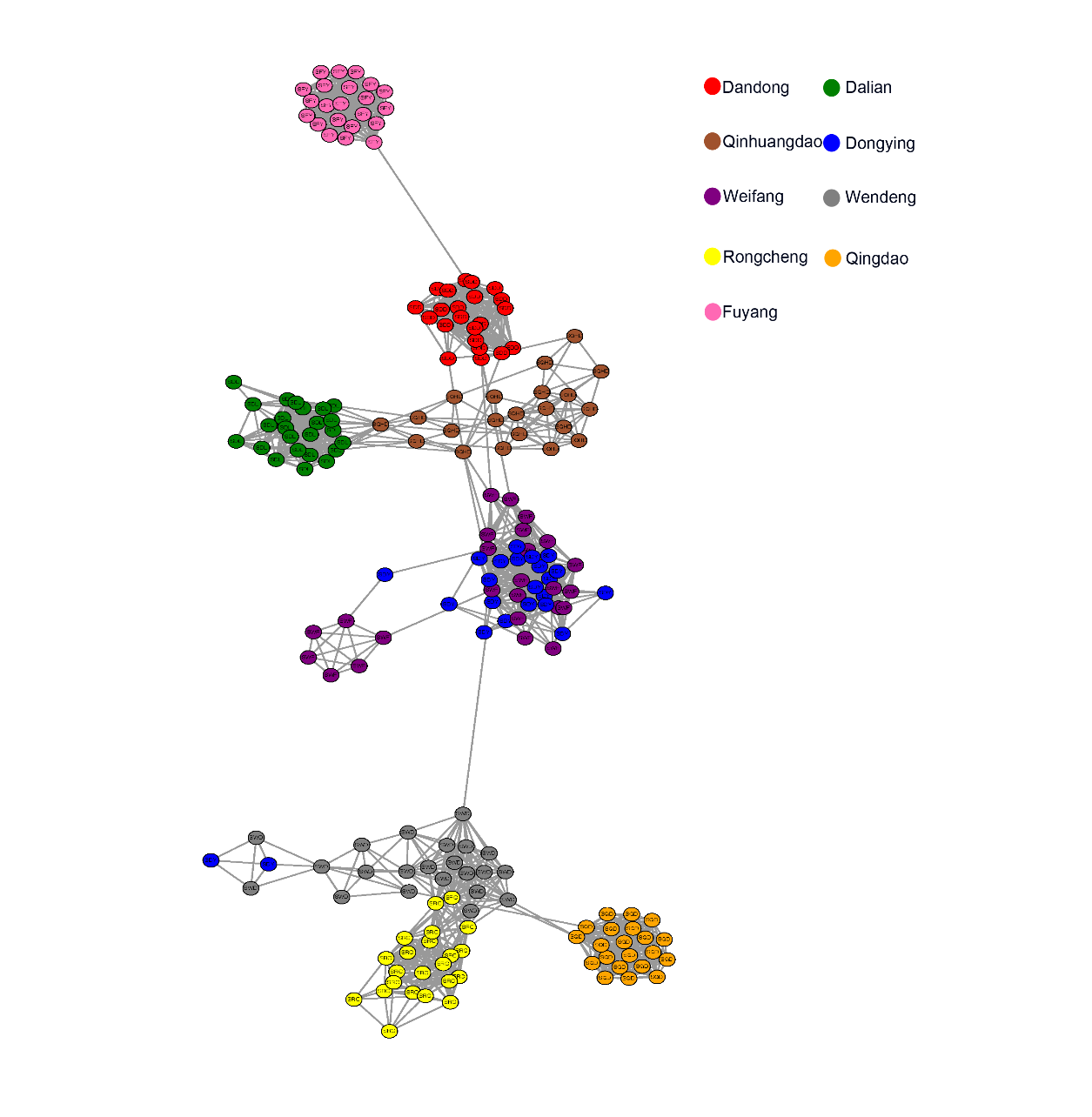


Fig. S3 Plot of BayeScan result, dots in red were outlier SNPs putatively under selection.


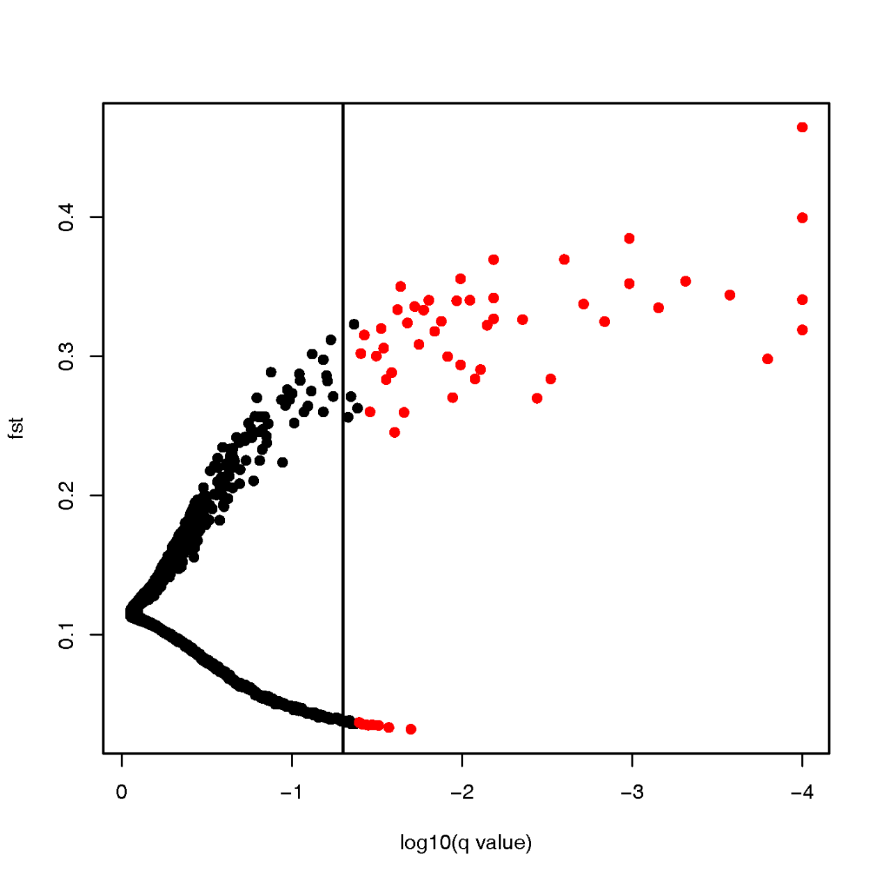


Fig. S4 Venn diagram for Bayenv results using four environmental variants. Lon: longitude, Lat: latitude, Annual: annual mean water temperature, Catadromous: mean water temperature during the catadromous period of *T. fasciatus*. Figure was generated by Venny 2.1.0 (http://bioinfogp.cnb.csic.es/tools/venny/index.html).


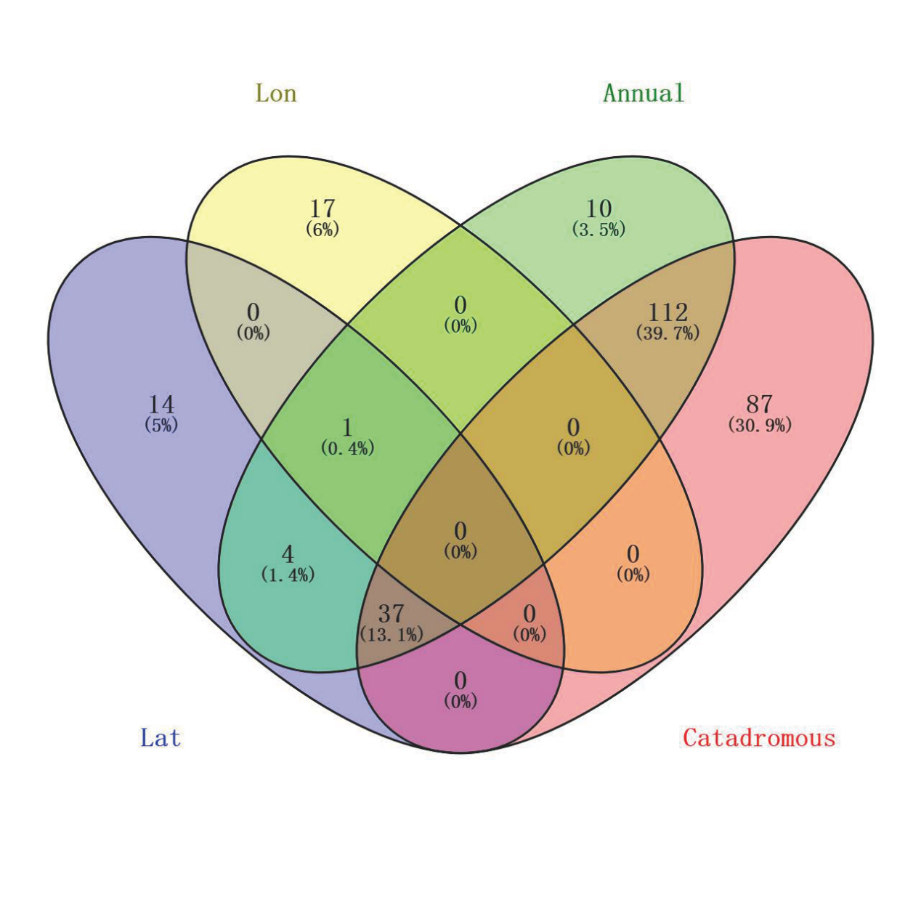


# Tables

Table S1. Sampling information for nine sites of *T. fasciatus*.

| Site | Code | Samples | Latitude | Longitude | Year |
| --- | --- | --- | --- | --- | --- |
| Dandong | SDD | 22 | 39.78 | 124.34 | 2014 |
| Dalian | SDL | 24 | 39.68 | 121.48 | 2016 |
| Qinhuangdao | SQHD | 21 | 39.85 | 119.6 | 2017 |
| Dongying | SDY | 23 | 37.74 | 119.36 | 2013 |
| Weifang | SWF | 25 | 37.12 | 119.48 | 2016 |
| Rongcheng | SRC | 22 | 37.35 | 122.64 | 2014 |
| Wendeng | SWD | 22 | 36.87 | 122.12 | 2015 |
| Qingdao | SQD | 21 | 36.15 | 120.25 | 2015 |
| Fuyang | SFY | 22 | 30.06 | 119.98 | 2014 |

Table S2. List of annotations for 74 outliers with GO IDs.

| Locus | Ensemble ID | GO IDs |
| --- | --- | --- |
| 102766 | ENSGACG00000012521.1 | F:GO:0004181; P:GO:0006508; C:GO:0016021 |
| 105321 | ENSGACG00000017791.1 | P:GO:0008360; C:GO:0016020 |
| 108288 | ENSGACG00000003007.1 | F:GO:0004497; F:GO:0005506; F:GO:0016705; F:GO:0020037; P:GO:0055114 |
| 110151 | ENSGACG00000005328.1 | F:GO:0000166; F:GO:0004422; C:GO:0005737; P:GO:0006166; P:GO:0032264; F:GO:0046872; F:GO:0052657 |
| 120146 | ENSGACG00000010573.2 | C:GO:0016020 |
| 123195 | ENSGACG00000017477.1 | F:GO:0003723; F:GO:0004000; P:GO:0006396 |
| 123508 | ENSGACG00000002036.1 | F:GO:0000049; F:GO:0004826; F:GO:0005524; C:GO:0005737; P:GO:0006432 |
| 129843 | ENSGACG00000017535.1 | F:GO:0003723 |
| 131986 | ENSGACG00000020188.1 | C:GO:0005887; P:GO:0007155; P:GO:0007399 |
| 134842 | ENSGACG00000013491.1 | F:GO:0005509 |
| 135459 | ENSGACG00000011082.1 | C:GO:0016021 |
| 162220 | ENSGACG00000015966.1 | F:GO:0004114; P:GO:0007601; F:GO:0030553 |
| 24281 | ENSGACG00000019743.1 | C:GO:0016021 |
| 26677 | ENSGACG00000000228.1 | F:GO:0005488; P:GO:0005975; F:GO:0016798 |
| 2895 | ENSGACG00000006703.1 | F:GO:0003735; C:GO:0005840; P:GO:0006412 |
| 29069 | ENSGACG00000000505.1 | F:GO:0004571; F:GO:0005509; P:GO:0008152; C:GO:0016020 |
| 29206 | ENSGACG00000000848.1 | C:GO:0016021 |
| 32238 | ENSGACG00000016504.1 | P:GO:0007094 |
| 34946 | ENSGACG00000000792.2 | F:GO:0003779; P:GO:0048747 |
| 3586 | ENSGACG00000018099.1 | C:GO:0016020 |
| 38311 | ENSGACG00000011082.1 | C:GO:0016021 |
| 40350 | ENSGACG00000006056.1 | F:GO:0046872; P:GO:0048701 |
| 42632 | ENSGACG00000000607.1 | F:GO:0004672; F:GO:0005524; P:GO:0006468 |
| 46965 | ENSGACG00000011082.1 | C:GO:0016021 |
| 46984 | ENSGACG00000001062.1 | C:GO:0005942; P:GO:0007165; F:GO:0035014; P:GO:0043551 |
| 47807 | ENSGACG00000014567.1 | C:GO:0016020 |
| 49632 | ENSGACG00000006539.1 | F:GO:0005509 |
| 50227 | ENSGACG00000003485.1 | F:GO:0004672; F:GO:0005524; P:GO:0006468 |
| 50809 | ENSGACG00000019985.1 | F:GO:0004198; F:GO:0005509; C:GO:0005622; P:GO:0006508; P:GO:0051493 |
| 51848 | ENSGACG00000013054.1 | F:GO:0000166; F:GO:0005515; P:GO:0006810; C:GO:0030176; F:GO:0042626 |
| 5749 | ENSGACG00000018488.1 | C:GO:0016021; F:GO:0022857; P:GO:0055085 |
| 57751 | ENSGACG00000004001.1 | C:GO:0005622; P:GO:0035556; F:GO:0046872 |
| 5849 | ENSGACG00000003464.1 | P:GO:0007010; F:GO:0051015 |
| 58561 | ENSGACG00000020188.1 | C:GO:0005887; P:GO:0007155; P:GO:0007399 |
| 61184 | ENSGACG00000002703.1 | F:GO:0004252; P:GO:0006508; F:GO:0070008 |
| 62808 | ENSGACG00000015006.1 | F:GO:0005509; F:GO:0005544; P:GO:0007268; P:GO:0007420; P:GO:0017158; P:GO:0035264 |
| 66531 | ENSGACG00000014362.1 | F:GO:0003810; P:GO:0018149 |
| 6830 | ENSGACG00000014360.1 | F:GO:0003676 |
| 68604 | ENSGACG00000012045.1 | F:GO:0003824; P:GO:0008152 |
| 70822 | ENSGACG00000004834.2 | F:GO:0003676; F:GO:0003700; P:GO:0006355; P:GO:0007224; P:GO:0009952; P:GO:0009953; P:GO:0048568; P:GO:0050767 |
| 8430 | ENSGACG00000009466.1 | C:GO:0005622; P:GO:0035556 |
| 85430 | ENSGACG00000015966.1 | F:GO:0004114; P:GO:0007601; F:GO:0030553 |
| 86486 | ENSGACG00000013609.2 | F:GO:0004930; P:GO:0007186; C:GO:0016020 |
| 9070 | ENSGACG00000011082.1 | C:GO:0016021 |
| 94482 | ENSGACG00000019893.1 | F:GO:0005488 |
| 109026 | ENSGACG00000014617.1 | F:GO:0003677; F:GO:0003700; P:GO:0008283; P:GO:0030154; P:GO:0045892 |
| 112127 | ENSGACG00000014731.1 | F:GO:0003712; P:GO:0006357; C:GO:0016592 |
| 113150 | ENSGACG00000004241.1 | F:GO:0005096; P:GO:0043547 |
| 118675 | ENSGACG00000003486.1 | C:GO:0016021 |
| 119085 | ENSGACG00000011250.1 | F:GO:0003676 |
| 124422 | ENSGACG00000019635.1 | C:GO:0005622; F:GO:0008270 |
| 133556 | ENSGACG00000007686.1 | C:GO:0016021 |
| 17076 | ENSGACG00000009661.1 | C:GO:0016021 |
| 18315 | ENSGACG00000012363.1 | F:GO:0003924; F:GO:0005509; F:GO:0005525; P:GO:0007005; C:GO:0031307 |
| 29871 | ENSGACG00000010073.1 | C:GO:0005737; F:GO:0016810; P:GO:0070997; P:GO:0097485 |
| 31156 | ENSGACG00000013795.1 | C:GO:0016021; P:GO:0030223; P:GO:0030224; P:GO:0030225; P:GO:0035162; P:GO:0060216; P:GO:1901534 |
| 32612 | ENSGACG00000017787.2 | F:GO:0003723 |
| 33633 | ENSGACG00000004385.1 | F:GO:0004367; P:GO:0005975; C:GO:0009331; F:GO:0042803; P:GO:0046168; F:GO:0051287; P:GO:0055114 |
| 37123 | ENSGACG00000008940.1 | P:GO:0009987; F:GO:0016491; P:GO:0055114 |
| 37673 | ENSGACG00000000321.1 | F:GO:0005548; C:GO:0005622; P:GO:0015914 |
| 39979 | ENSGACG00000001887.1 | F:GO:0003677; F:GO:0003887; C:GO:0005634; P:GO:0006281; F:GO:0046872; P:GO:0071897 |
| 4064 | ENSGACG00000002383.1 | C:GO:0016021 |
| 40915 | ENSGACG00000014072.1 | C:GO:0016021; P:GO:0030213; F:GO:0050501 |
| 46288 | ENSGACG00000000743.1 | P:GO:0000413; F:GO:0003755; C:GO:0016021; P:GO:0071391 |
| 60096 | ENSGACG00000001225.1 | C:GO:0005622; P:GO:0006886 |
| 63121 | ENSGACG00000011879.1 | C:GO:0016021; P:GO:0055085 |
| 64130 | ENSGACG00000018400.1 | P:GO:0001539; F:GO:0003777; C:GO:0005858; P:GO:0007018; F:GO:0016887 |
| 65678 | ENSGACG00000017791.1 | P:GO:0008360; C:GO:0016020 |
| 67049 | ENSGACG00000015487.1 | C:GO:0005743; P:GO:0030150; F:GO:0051087 |
| 69728 | ENSGACG00000020088.1 | F:GO:0004674; F:GO:0005524; C:GO:0005622; P:GO:0006468; P:GO:0043491 |
| 73378 | ENSGACG00000016595.1 | F:GO:0046872 |
| 7573 | ENSGACG00000005132.1 | C:GO:0016021 |
| 9080 | ENSGACG00000017724.1 | P:GO:0007155; C:GO:0016021; P:GO:0046718 |
| 99113 | ENSGACG00000015789.2 | P:GO:0001837; C:GO:0016021; P:GO:0030001; P:GO:0042074; F:GO:0046873; P:GO:0055085 |

|  | AU1 | AU2 | AU3 | AU4 | AU5 | AU6 |
| --- | --- | --- | --- | --- | --- | --- |
| AU1 |  |  |  |  |  |  |
| AU2 | 0.1475 |  |  |  |  |  |
| AU3 | 0.2267 | 0.2243 |  |  |  |  |
| AU4 | 0.4461 | 0.3908 | 0.4012 |  |  |  |
| AU5 | 0.5293 | 0.4447 | 0.4510 | 0.3312 |  |  |
| AU6 | 0.5694 | 0.5403 | 0.5308 | 0.6693 | 0.7518 |  |

Table S3. Pairwise *F*_ST_ values among six adaptive units (AUs) of *T. fasciatus*. AU1: Dandong; AU2: Dalian & Qinhuangdao; AU3: Dongying & Weifang; AU4: Rongcheng & Weifang; AU5: Qingdao; AU6: Fuyang.

References:

Shannon P, et al. 2003. Cytoscape: A Software Environment for Integrated Models of Biomolecular Interaction Networks. Genome Res. 13: 2498-2504.
